# The effect of A2E on lysosome membrane permeability during blue light-induced human RPEs apoptosis

**DOI:** 10.1186/s12886-022-02464-1

**Published:** 2022-05-31

**Authors:** Yan Xu, Dan Li, Gang Su, Shanjun Cai

**Affiliations:** 1 Department of Ophthalmology, Affiliated Hospital of Zunyi Medical University, No.149 Dalian Road, Zunyi, 563000 Guizhou China; 2 Department of Ophthalmology, HwaMei Hospital, University of Chinese Academy of Sciences, Ningbo No.2 Hospital, Ningbo, Zhejiang China; 3 Department of Ophthalmology, The People’s Hospital of Longchang, Neijiang, Sichuan China; 4 Guizhou Eye Hospital, Zunyi, Guizhou China; 5 Guizhou Provincial Branch of National Eye Disease Clinical Medicine Research Center, Zunyi, Guizhou China; 6 Special Key Laboratory of Ocular Diseases of Guizhou Province, Zunyi Medical University, Zunyi, Guizhou China

**Keywords:** Apoptosis, Lysosomal membrane permeability (LMP), N-retinyl-N-retinylidene ethanolamine (A2E), Blue light, Retinal pigment epithelium cells (RPEs), Apoptosis, Age-related macular degeneration (AMD)

## Abstract

**Background:**

To investigate the effect of N-retinyl-N-retinylidene ethanolamine (A2E) on lysosome membrane permeability (LMP) during blue light-induced human retinal pigment epithelium cells (RPEs) apoptosis.

**Methods:**

By building an A2E and blue light irradiation inducing RPEs damage model, the CCK-8 assay was used to detect RPEs viability loaded with different concentrations of A2E after different culturing time to determine the optimum A2E loading concentration. And the RPEs fluorescence intensity changes were observed by fluorescence microscopy loaded with different concentration of A2E. The RPEs were divided into four groups randomly: control group, A2E-loaded group, blue light irradiation group, and A2E-loaded + blue light irradiation group. Annexin V-FITC/PI and TUNEL/DAPI methods were used to detect RPEs apoptotic rate. Laser scanning confocal microscopy (LSCM) was used to observe RPEs LMP changes stained by acridine orange (AO) method.

**Results:**

The CCK-8 result showed a downward trend in cells viability of RPEs loaded with increasing concentration of A2E and extending culturing time. The optimum A2E loading concentration was determined at 25 μmol/L. With increasing A2E loading concentrations, the intensity of fluorescence in RPEs decreased gradually. The RPEs apoptotic rate in blue light irradiation + A2E-loaded group was significantly higher than those in other three groups detected by Annexin V-FITC/PI method, which was similar to TUNEL/DAPI’s result. After AO staining, cytoplasmic and nucleolar RNAs emits green fluorescence; lysosomes emit red fluorescence. Through the interference of A2E and blue light on RPEs, red fluorescent leakage from the lysosomes (means LMP increasing) can be observed. The mean red fluorescence intensity was chosen as the statistics indicator to estimate LMP change in RPEs cultured in vitro. Compared with the control group, the red fluorescence intensity decreased in A2E-loaded group, blue light irradiation group, and blue light irradiation + A2E-loaded group. Meanwhile, the mean red fluorescence intensity in blue light irradiation + A2E-loaded group was the lowest.

**Conclusions:**

Both A2E-loaded and blue light irradiation could induce human RPEs apoptosis, and the two factors had a synergistic effect. In addition, both A2E and blue light can lead to LMP increasing, which indicated LMP change might be the upstream part in inducing mitochondrion-dependent apoptotic pathway. These data provided evidence that A2E as the most important auto-fluorescence substance in lipofuscin is an initiator of blue light-mediated damage of RPEs and participate in pathogenesis of retinal degenerative diseases in humans.

## Background

It was generally agreed both visible light and ultraviolet light irradiation could induce photochemical lesions to retina which was closely related to some retinal degenerative diseases like age-related macular degeneration (AMD) [1]. The apoptosis of retinal pigment epithelium cells (RPEs) was the important mechanism during AMD disease development and might happened at the onset of the occurring [2]. Some research had verified that aging of cultured RPEs relied on blue light damage, lipofuscin formation, and oxidative reactions [3].

Blue light irradiation-induced RPEs apoptosis based on the signaling pathway and oxidative stress. Sparrow JR et al. considered that blue light illumination of RPEs initiated a cell death program that was executed by a proteolytic caspase cascade and that was regulated by Bcl-2 [4]. Our team had verified in previous experiments that at the early stage of blue light irradiation, the mitochondrial membrane potential declined, the cytochrome C released, and the expression of caspase-9 enhanced, accompanying with the downregulation of Bcl-2, Bcl-xl, and upregulation of Bax expression. These features indicated that blue light irradiation could cause mitochondrial membrane damage. Meanwhile, the mitochondrial apoptotic pathway participated in blue light irradiation-induced human RPEs apoptosis cultured in vitro [5, 6].

Lipofuscin (LF) was the metabolic product of retinaldehyde deposited in the lysosomes of RPEs, which was produced by phagocytosis of RPEs of the photoreceptor outer segment (POS) [7, 8]. A2E was the major fluorophore of LF with autofluorescence and phototoxicity [9–11]. Excess accumulation of LF in RPEs could advance the sensibility of RPEs to blue light [12]. Some research had verified that after human RPEs cultured in vitro phagocytized LF and subsequently exposed to 480 ± 20-nm blue light, A2E could produce substantial reactive oxygen species (ROS) such as O^2−^, H_2_O_2_, ·OH, HO_2_·by epoxidation then induced RPEs apoptosis or damage [2, 13]. These factors were considered to contribute to the onset of retinal degenerative diseases including Stargardt disease and AMD [14, 15].

Mitochondria were the main organelles produced ROS, and the effective range of ROS was limited. Therefore, lysosomes situated around mitochondria would be more vulnerable to ROS and occurred lysosomal membrane permeability (LMP) change. Lysosomes were commonly recognized as “suicide bags” [16]. They could induce cells apoptosis or necrosis by releasing cathepsins from lysosomes to cytoplasm when lysosomal membrane damage happened or LMP increased [17]. More articles further indicated that the changes in LMP were relative to blue light irradiation and A2E. With A2E effects, RPE apoptosis induced by blue light irradiation would be more serious, but the real mechanism had not been elucidated. To this end, using A2E synthesized in vitro and loaded on RPEs, we built a model of A2E-loaded and blue light irradiation inducing RPEs apoptosis in vitro. This cell cultured model could imitate the pathological change of RPEs in AMD [18, 19]. Furthermore, we discussed the changes in LMP when blue light irradiation inducing apoptosis in RPEs loaded with A2E, which could provide further experimental evidence for the mechanism of damage.

## Methods

### Human RPEs culture

According to Li’s reported method [20], the ARPE-19 human RPE cell line (American Type Culture Collection, Manassas, VA) was utilized in this study. Every parallel control trial used RPEs in the same generation, and cells grew into the log phase were harvested.

### A2E synthesis

According to Feng’s experiment [21], A2E was prepared from 100 mg all-trans-retinal (Sigma Aldrich, St. Louis, MO, USA) and 9.5 mg ethanolamine (Solarbio, Beijing, China) in 2 ml ethanol. A2E was stored in dimethyl sulfoxide (DMSO) at 25 mM under − 80 °C in the dark.

### RPEs viability detected by CCK-8 assay

When RPEs in the log phase, cells were inoculated into 96-well plates (5 × 10^4^ cells/ml). Five groups were set for this experiment, 3 trial groups (RPEs with A2E concentration at 10, 25, and 50 μmol/L respectively), a control group (RPEs without A2E), and a blank control group (only had equivalent complete medium and CCK-8 when detection). Each group had 5 duplications. The plates were cultured in an incubator with 5% CO_2_ at 37 °C for 12, 24, 48, or 72 h separately. After culturing, 100 μL complete medium with a 10% concentration of CCK-8 (Dojindo, Kumamoto, Japan) was added into each well, then the plates were cultured in an incubator for another 3 h. Microplate reader (Thermo, IR, USA) was used to detect the absorbance, and cell viability was evaluated by the integral absorbance (IA) value according to the following formula: (trial group − blank control group) IA/(control group − blank control group) IA × 100%. The assay was repeated at least three times.

### The intracytoplasmic fluorescence intensity changes of RPEs loaded with different A2E concentration observed by fluorescence microscopy

Coverslips were placed in 4 wells chosen from a six-well plate. RPEs were inoculated into these wells (1 × 10^5^ cells/ml) fulfilled with complete medium and cultured in an incubator with 5% CO_2_ at 37 °C for 48 h. After 48 h incubation, the complete medium was replaced by a non-blood serum medium and the plate was cultured for another 24 h. After culturing, the prepared A2E was added into each well reaching a final A2E concentration at 0, 10, 25, and 50 μmol/L respectively. Then, the plate was cultured in the incubator previously mentioned for extra 2 h. After the coverslips were fixed by 4% paraformaldehyde for 20 min, the intracytoplasmic fluorescence intensity change was observed by fluorescence microscopy (Ex/Em = 460–550 nm/510–560 nm; Leica, Wetzlar, Germany). All the images were taken at the same condition. Five views from every coverslip and 20 cells from every view were chosen randomly. Fluorescence microscopy (Leica, Wetzlar, Germany) was used to quantify fluorescence intensity and the result was expressed in mean fluorescence intensity. The assay was repeated at least three times.

### Build the RPEs apoptotic model loaded with A2E exposing to blue light irradiation

RPEs were divided into four groups randomly: control group (not load with A2E and without blue light irradiation), A2E-loaded group, blue light irradiation group, and A2E-loaded + blue light irradiation group. The optimum A2E loading concentration was determined by previous trial. Blue light irradiation group and A2E-loaded + blue light irradiation group were exposed to medical blue light irradiation. The control group and A2E-loaded group that did not need to receive blue light irradiation were covered with silver paper. All four groups were placed in a home-made blue light equipment with a 20 W, 450–500 nm (2000 ± 500) lux medical blue light lamp, and exposed to the blue light at 37 °C for 6 h. After 6 h irradiation, all four groups were cultured to an optimum terminal time which determined by previous trial and continuing cultured in an incubator with 5% CO_2_ at 37 °C for 24 h [20].

### RPEs apoptotic rate detection

The Annexin V-FITC/PI method was used to detect RPEs apoptotic rate. Based on Cai’s research [5], RPEs were inoculated into six-well pates and cultured according to the RPEs apoptotic model loaded with A2E exposing to blue light irradiation mentioned before. After culturing, cells were collected and washed by PBS twice then transferred into a 15-ml centrifuge tube. The RPEs were resuspended in 200 μL binding buffer with 10 μL of FITC-Annexin V (Solarbio, Beijing, China) and 10 μL of PI (Solarbio, Beijing, China) added and were blended gently. Subsequently, the cells were incubated at 4 °C for 30 min in dark, followed by the supplement of 300 μL binding buffer into the tube. Finally, flow cytometer (BD FACSVerse, San Jose, CA, USA) using quest software was performed immediately. The experiment was repeated three times.

The TUNEL and DAPI-stained method was used to determine the percentage of RPE apoptotic nuclei. Coverslips were placed in six-well pates and RPEs were inoculated into these wells (1 × 10^5^ cells/ml) fulfilled with complete medium. The RPEs apoptotic model loaded with A2E exposing to blue light irradiation was built in accordance with the above method. For staining, the cultures were fixed by 4% paraformaldehyde for 20 min, permeabilized with 0.1% Triton X-100 in PBS (5 min, 4 °C; Solarbio, Beijing, China), incubated in terminal deoxynucleotidyl transferase (TdT) together with FITC-dUTP (37 °C, 60 min; Solarbio, Beijing, China), and then stained with 4′,6-diamidino-2-phenylindole (DAPI; Solarbio, Beijing, China). The result was visualized by fluorescence microscopy (Ex/Em = 450–500 nm/515–565 nm, × 200 objective; Leica, Wetzlar, Germany) and counted from digital images. RPEs apoptotic rate in one view = TUNEL stained positive cells number in one view/total cells number in one view × 100%. Five views with positive cells were chosen randomly, and the average value of RPEs apoptotic rate was calculated. The experiment was repeated three times.

### RPEs intracellular lysosome membrane permeability changes detected by LSCM

In order to observe intracellular lysosome membrane permeability (LMP) change, acridine orange (AO) was used to stain living RPEs cultured in vitro. RPEs crawling slides were prepared and divided into four groups as mentioned above. The slides were cultured in six-well plates according to the A2E-loaded and blue light irradiation model as previously described. After A2E loading and 6 h irradiation followed with 24 h of culture, 0.01% AO (Solarbio, Beijing, China) solution 500 μL was added into each group. Then, the slides were cultured for another 15 min with 5% CO_2_ at 37 °C, washed with PBS and mounted by 10% glycerin. Laser scanning confocal microscopy (LSCM; Leica, Wetzlar, Germany) was used to observe intracellular fluorescence intensity changes and images were collected. In these images, AO binding to cytoplasmic and nucleolar RNAs emits green fluorescence and accumulates in acidic vesicles like lysosomes, emits red fluorescence. The mean red fluorescence intensity was chosen as the statistics indicator to estimate RPEs LMP change [12]. All the images were taken at the same condition of exposure time and exposure intensity. 5 views from every slide and 20 cells from every view were chosen randomly. Leica confocal software (LCS Lite; Leica, Wetzlar, Germany) was used to quantify fluorescence intensity and the result was expressed in mean fluorescence intensity. The assay was repeated at least three times.

### Statistical analysis

Statistical analysis was performed using SPSS 17.0 software (IBM, Armonk, NY, USA), and the date was presented as the mean ± standard deviation (SD). GraphPad Prism 6.0 (GraphPad Software, La Jolla, CA, USA) was used to perform statistical graphs. One-way analysis of variance (ANOVA) was applied, comparing the population mean in each group. If there were significant difference, the least significant difference-test (LSD-t) was used for comparisons. *α* = 0.05 and *P* < 0.05 were considered significant.

## Results

### Analysis of RPEs viability detected by CCK-8 assay

**Fig. 1:**
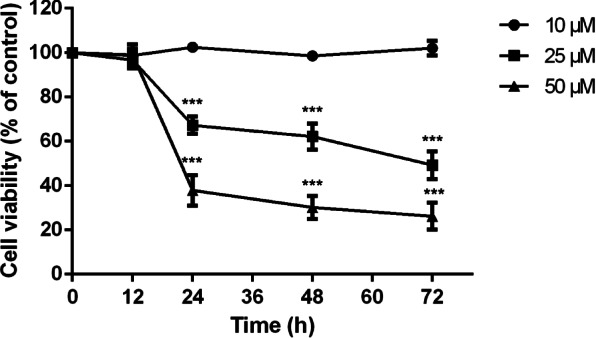
Cell viability of RPEs loaded with A2E concentration at 10, 25, and 50 μmol/L and cultured for 12, 24, 48, and 72 h detected by CCK-8 assay. Cell viability was evaluated by the integral absorbance (IA) value formula: (trial group—blank control group) IA/(control group—blank control group) IA × 100%. The cell viability of RPEs decreased as A2E concentration increasing or extending culturing time. When the concentration of A2E reached to 25 μmol/L or 50 μmol/L, there were significant differences in the cell viability between RPEs after culturing for 24, 48, or 72 h from the control group. Mean ± SD., *N* = 3. Statistical analysis: ***P < 0.001 as compared with the control group; one-way ANOVA and LSD-t

RPEs were cultured with A2E at 10, 25, or 50 μmol/L for 12, 24, 48, or 72 h. The viability of RPEs decreased as A2E concentration increasing or extending culturing time (Fig. 1). When RPEs were cultured for 12 h, for the cells viability, there was no significant difference between trial groups (A2E concentration at 10, 25, and 50 μmol/L) and the control group (*P*=0.672, 0.256, 0.767). When the concentration of A2E reached to 10 μmol/L, the cell viability was not significantly different in RPEs after culturing for 24, 48, or 72 h from the control group (*P*=0.295, 0.695, 0.589). However, when the concentration of A2E reached to 25 μmol/L or 50 μmol/L, there were significant differences in the cells viability between RPEs after culturing for 24, 48, or 72 h from the control group (*P*< 0.001, 0.001, 0.001, *P*< 0.001, 0.001, 0.001).

### The intracytoplasmic fluorescence intensity changes of RPEs loaded with different A2E concentration observed by fluorescence microscopy

**Fig. 2:**
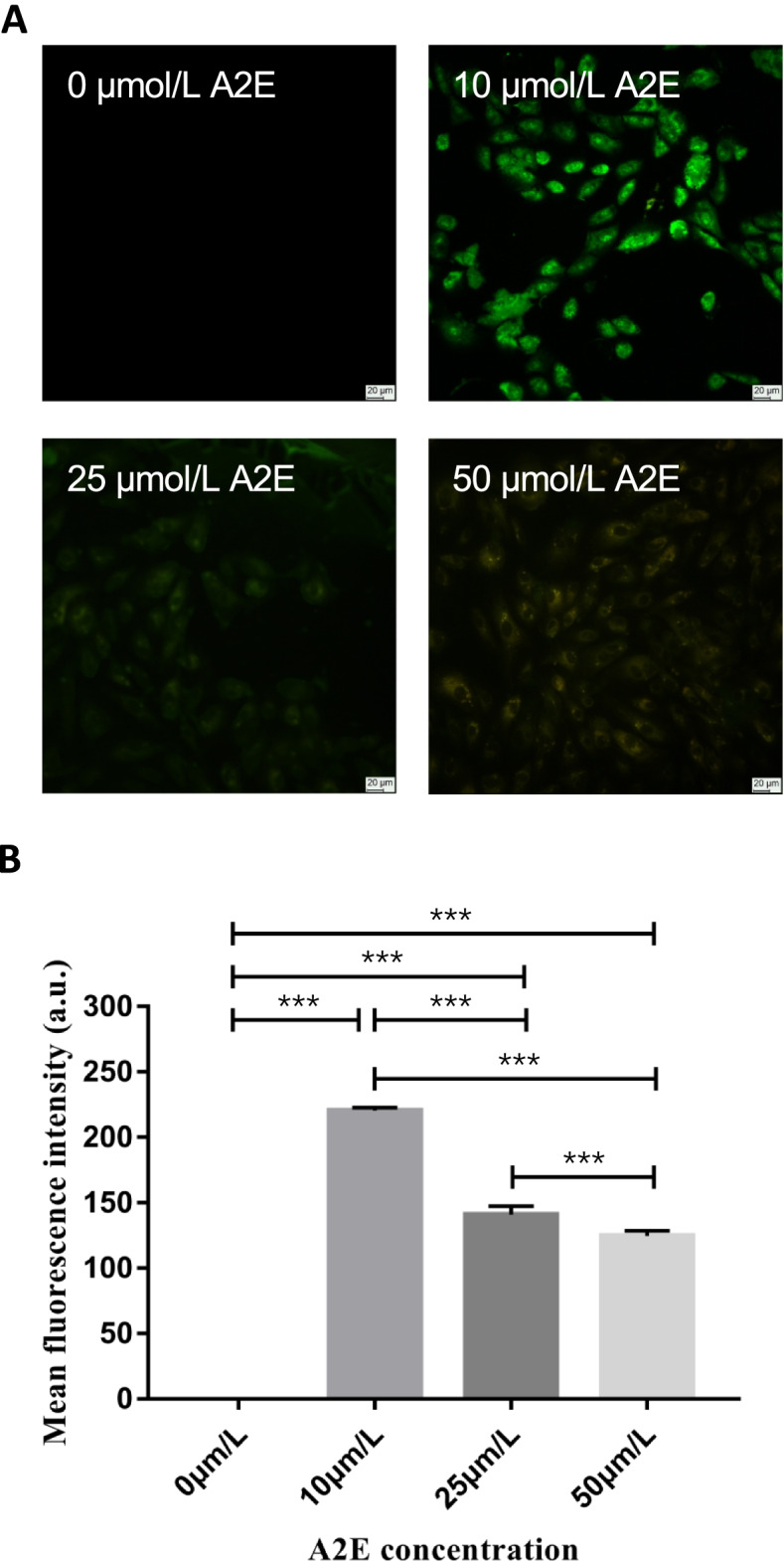
The RPEs loaded with A2E concentration at 0, 10, 25, and 50 μmol/L separately and cultured for 2 h observed by fluorescence microscope. **A** The image showed there was no intracytoplasmic fluorescence in RPEs cultured with A2E concentration at 0 μmol/L. And the intensity of intracytoplasmic fluorescence in RPEs decreased gradually with the A2E concentration increasing (× 200 objective, Scale bar: *20 μm*). **B** The comparison of mean fluorescence intensity in RPEs loaded with A2E concentration at 0, 10, 25, and 50 μmol/L. Mean ± SD., *N* = 3. Statistical analysis: ***P < 0.001; one-way ANOVA and LSD-t

The RPEs fluorescence results observed by fluorescence microscopy showed that there was no intracytoplasmic fluorescence in RPEs cultured with A2E concentration at 0 μmol/L. Since the A2E concentration increasing, the intensity of intracytoplasmic fluorescence in RPEs decreased gradually (Fig. 2A).

The mean fluorescence intensity of RPEs loaded with A2E at 0, 10, 25, or 50 μmol/L were 220.254 ± 2.427, 140.880 ± 6.542, and 124.556 ± 4.011, respectively, and the difference was significant (*F* = 1537.545, *P* < 0.001). Among 0, 10, 25, or 50 μmol/L A2E groups, the mean fluorescence intensity in 0 μmol/L A2E group was the lowest with significant difference (*P* < 0.001, 0.001, 0.001). The mean fluorescence intensity in 10 μmol/L A2E group was higher than 25 or 50 μmol/L A2E groups with significant difference (*P* < 0.001, 0.001). And the mean fluorescence intensity in 25 μmol/L A2E group was little more than the value in 50 μmol/L A2E group with significant difference (*P* < 0.001) (Fig. 2B).

### RPEs apoptosis detection

**Fig. 3:**
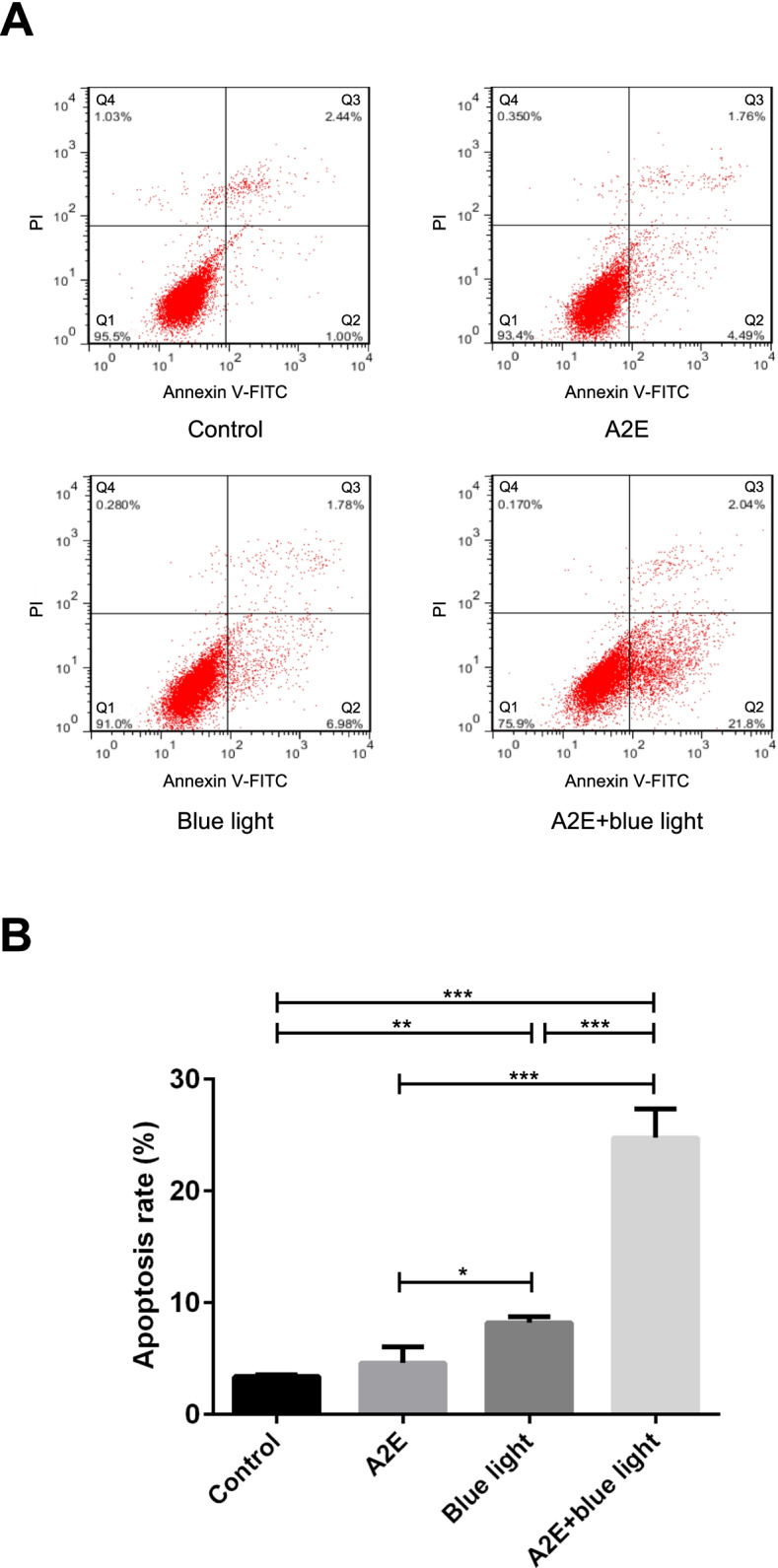
The RPEs apoptotic rate in control group, A2E group, blue light group, and A2E + blue light group detected by flow cytometer used Annexin V-FITC/PI double staining method. **A** In this image: Q1: The left lower quadrant was Annexin V-FITC (-) and PI (-), represented viable cells. Q2: The right lower quadrant was Annexin V-FITC ( +) and PI (-), represented viable apoptotic cells. Q3: The right upper quadrant was Annexin V-FITC ( +) and PI ( +), represented non-viable apoptotic cells. Q4: The left upper quadrant was Annexin V-FITC (-) and PI ( +), represented non-viable necrotic cells. The sum of Q2 and Q3 presented apoptotic RPEs. **B** The comparison of RPEs apoptotic rate in different groups detected by Annexin V-FITC/PI staining method analyzed by flow cytometry. Mean ± SD., *N* = 3. Statistical analysis: *P < 0.05, **P < 0.01, ***P < 0.001; one-way ANOVA and LSD-t

The results of the Annexin V-FITC/PI double staining method indicated that the apoptotic rate of RPEs in the control group, the A2E-loaded group, the blue light irradiation group, and the A2E-loaded + blue light irradiation group were 3.39 ± 0.15%, 4.61 ± 1.44%, 8.21 ± 0.52%, and 24.77 ± 1.49%, respectively, with a significant difference (*F* = 130.292, *P* < 0.001). The apoptotic rate in the blue light irradiation group and the A2E-loaded + blue light irradiation group were significantly above the control group (*P* = 0.004, *P* < 0.001). However, for the apoptotic cell rate, there was no significant difference between the A2E-loaded group and the control group (*P* = 0.348). Besides, among the A2E-loaded group, the blue light irradiation group and the A2E-loaded + blue light irradiation group, the apoptotic rate in A2E-loaded + blue light irradiation group was significantly higher than the rate in the other two groups (*P* < 0.001, 0.001). The apoptotic rate in the blue light irradiation group was significantly higher than the rate in the A2E-loaded group (*P* = 0.019) (Fig. 3A and B).

**Fig. 4:**
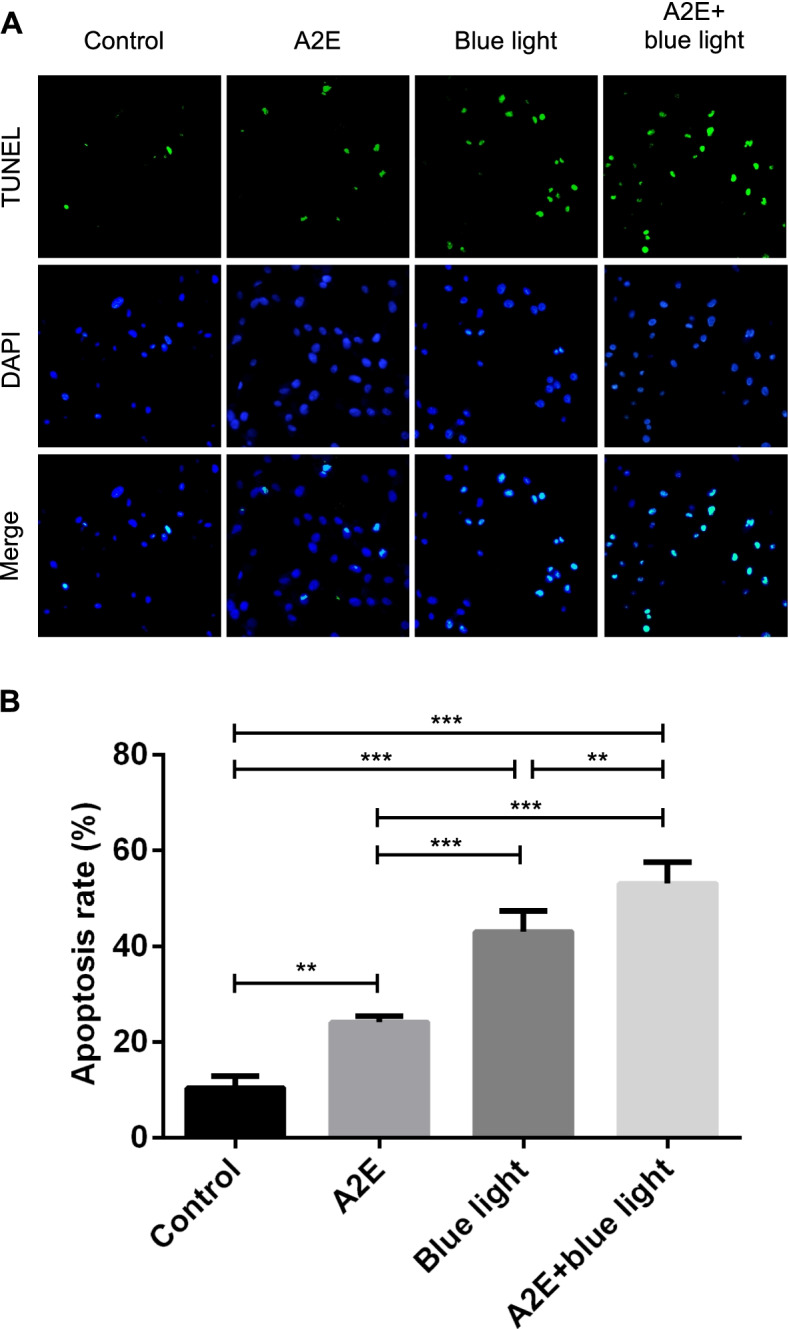
The RPEs apoptotic rate in control group, A2E group, blue light group, and A2E + blue light group observed by fluorescence microscope detected by TUNEL and DAPI-stained method. **A** The green fluorescence represented apoptotic RPE nuclei meanwhile the blue fluorescence represented all RPE nuclei. The apoptotic RPEs were stained as green/blue double fluorescence which was considered as TUNEL stained positive cells (× 200 objective, Scale bar: *20* μm). **B** The comparison of RPEs apoptotic rate in different groups detected by TUNEL and DAPI-stained method observed by fluorescence microscope. RPEs apoptotic rate calculation: RPEs apoptotic rate = TUNEL stained positive cells number/total cells number × 100%. Mean ± SD., *N* = 3. Statistical analysis: **P < 0.01, ***P < 0.001; one-way ANOVA and LSD-t

After TUNEL and DAPI staining, the nuclei of apoptotic RPEs were stained as green fluorescence, and the nuclei of all the RPEs were stained as blue fluorescence. The RPEs stained as green/blue double fluorescence were selected as TUNEL stained positive cells (Fig. 4A). The apoptotic rate of RPEs in the control group, the A2E-loaded group, the blue light irradiation group, and the A2E-loaded + blue light irradiation group were 10.40 ± 2.46%, 24.07 ± 1.17%, 43.00 ± 4.41%, and 53.00 ± 4.50%, respectively, with a significant difference (*F* = 92.423, *P* < 0.001). The apoptotic rate in the A2E-loaded group, the blue light irradiation group, and the A2E-loaded + blue light irradiation group were significantly higher than the rate in the control group (*P* < 0.001, 0.001, 0.001). Compared with blue light irradiation group and A2E-loaded group, the apoptotic rate in the A2E-loaded + blue light irradiation group was significantly higher (*P* = 0.007, *P* < 0.001). Moreover, the apoptotic rate in the blue light irradiation group was significantly higher than the rate in the A2E-loaded group (*P* < 0.001) (Fig. 4B).

### RPEs intracellular lysosome membrane permeability changes observed by LSCM

**Fig. 5:**
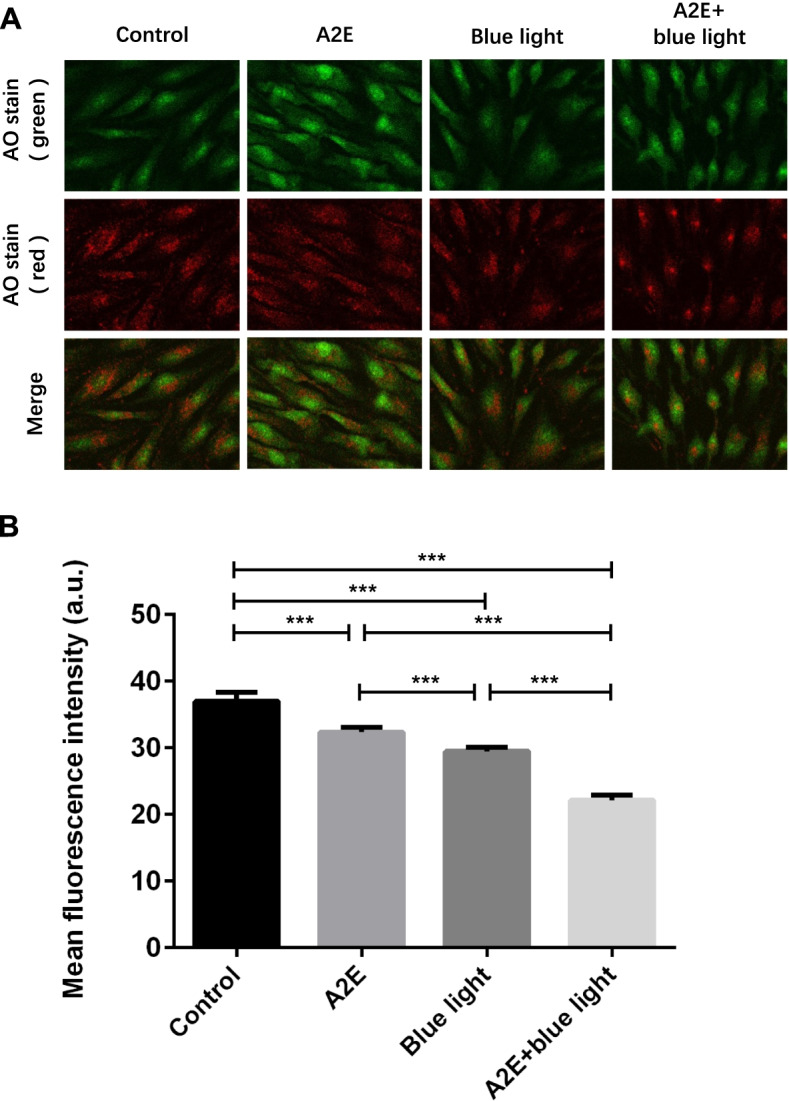
The fluorescence intensity in control group, A2E group, blue light group, and A2E + blue light group stained by AO analyzed by LCS Lite. **A** The image of living RPEs in different groups stained by AO and observed by LSCM (× 400 objective). In living RPEs cultured in vitro, AO binding to cytoplasmic and nucleolar RNAs emits green fluorescence and accumulates in acidic vesicles like lysosomes, emits red fluorescence. The mean red fluorescence intensity was chosen as the statistics indicator to estimate LMP change in RPEs cultured in vitro affected by A2E and blue light irradiation. **B** The comparison of mean fluorescence intensity (red) in different groups stained by AO analyzed by LCS Lite. Mean ± SD., *N* = 3. Statistical analysis: ***P < 0.001; one-way ANOVA and LSD-t

After AO stained living RPEs cultured in vitro, AO binding to cytoplasmic and nucleolar RNAs emits green fluorescence and accumulates in acidic vesicles like lysosomes, emits red fluorescence. Red fluorescent leakage from the lysosomes can be observed by LSCM in the A2E-loaded group, the blue light irradiation group, and the A2E-loaded + blue light irradiation group, which indicates LMP increasing (Fig. 5A). Thus, the mean red fluorescence intensity was chosen as the statistics indicator to estimate LMP change in RPEs cultured in vitro affected by A2E and blue light irradiation. The value was expressed as mean fluorescence intensity. The mean fluorescence intensity in the control group, the A2E-loaded group, the blue light irradiation group, and the A2E-loaded + blue light irradiation group were 37.00 ± 1.30, 32.37 ± 0.72, 29.48 ± 0.62, and 22.16 ± 0.73, respectively, and the difference was significant (*F* = 246.955, *P* < 0.001). Among those groups, the mean fluorescence intensity in the blue light irradiation group, the A2E-loaded group and the A2E-loaded + blue light irradiation group were lower than the value in the control group with significant difference (*P* < 0.001, 0.001, 0.001). The mean fluorescence intensity in the blue light irradiation group was below the value in the A2E-loaded group with significant difference (*P* < 0.001). The mean fluorescence intensity in the A2E-loaded + blue light irradiation group was less than the value in the blue light irradiation group and the A2E-loaded group with significant difference (*P* < 0.001, 0.001) (Fig. 5B).

## Discussion

A2E had been recognized as one of the risk factors of RPEs apoptosis [4]. It was composed of bimolecular all-trans-retinal and monomolecular ethanolamine [22]. The precursor of A2E was formed in the rodoutersegmeni (ROS); however, A2E itself existed in the phagolysosomes of RPEs [23]. In consequence, we inferred that A2E might be the initiation factor of RPEs apoptosis. Many experiments discussed the relationship between A2E and lysosomes and made a conclusion that during phagocytosis of lysosomes, autophagy might participate in the process of A2E accumulation [24, 25]. When A2E reached to the critical concentration, it could inhibit the function of the lysosomal proton pump, which led to substances inside the lysosomes leaking to the cytoplasm [8]. A2E also could destroy the DNA or mitochondrial membranes of RPEs. All these factors would induce RPEs apoptosis.

Absorbing a wide light spectrum, particularly visible blue light, was one of the key characteristics of A2E caused light injury of RPEs [26]. Sparrow suggested that A2E can exhibit cytotoxicity by photo-chemical damage and is an initiator of blue light-mediated damage of RPEs [2]. Under the condition of certain light irradiation and absorbing certain wavelengths of light, A2E could undergo photoisomerization. During the process of isomerization, it would release free radicals to change the structure and function of the cytomembrane or intracellular lysosomal membrane of RPEs. It could even affect the internal lysosomal enzyme activity directly then led to RPEs injury and dysfunction [27–29]. Therefore, a cell cultured model of A2E-loaded and blue light irradiation inducing RPEs apoptosis was constructed to simulate this pathological change.

Some researchers compared the content of A2E harvested from RPEs of 58- to 79-year-old healthy donors’ eyes with the content of intracellular A2E which produced by RPEs culturing with different concentrations of A2E in vitro and found the content of intracellular A2E in RPEs co-cultured with 10–25 μmol/L A2E in vitro was closed to the content of A2E in geriatric eyes’ RPEs (34–134 ng/10^5^) [8]. According to ours CCK-8 assay results (Fig. 1), RPEs loaded with A2E concentrations at 25 μmol/L or 50 μmol/L and cultured for 24 h compared with the control group, the result was significant difference. Considering the toxic effect of A2E to cells, A2E concentration at 25 μmol/L was chosen as the optimum concentration loaded with RPEs, which was similar to Zhang J’s research [30].

Sparrow JR et al. argued that A2E was the initiator during RPEs apoptosis [4, 31]. Blue light at a 480-nm wavelength could induce apoptosis of RPEs loaded with A2E. With A2E effects, the degree of RPEs’ injury would be more serious, which was similar to our experiment’s result [4, 12]. We use Annexin V-FITC/PI and TUNEL methods to detect RPEs apoptosis. The RPEs apoptotic rate in the A2E-loaded group, the blue light irradiation group, and the A2E-loaded + blue light irradiation group were higher than that in the control group. It elucidated both A2E and blue light irradiation could induce RPEs apoptosis, and the two factors had a synergistic effect (Figs. 3 and 4).

In addition, ROS could not only participating in cells apoptosis by increasing LMP but also injuring the lysosomal membrane directly [32]. When LMP is increasing, the cathepsin B, D etc., would be released from lysosomes to cytoplasm and spliced Bcl-2 family proteins like Bid, Bax, or Bak. Then, mitochondrial membrane permeability (MMP) would increase, which promoted mitochondrial cytochrome C released and activated caspase family cascade and finally induced cell apoptosis [33–35]. By this inference, LMP change was confirmed as a committed step in inducing mitochondrion-dependent apoptotic pathway [36, 37].

We used AO staining method to detect LMP change in RPEs loaded with A2E after blue light irradiation. AO was a lyotropic metachromatic fluorescent dye; AO stainability differs completely between fixed cells and cultured cells [38]. Living RPEs cultured in vitro have intact bio-membranes, including cytoplasmic, nuclear, and lysosomal membranes. AO diffuses through the cytoplasmic membrane and is retained in the cellular compartments with low pH, resulting in a red fluorescence of lysosomes when excited with a blue light. AO also intercalates with the cytoplasmic and nuclear RNA molecules and results in a diffuse green fluorescence within all cells [39], as the Fig. 5A control group showed. In contrast, apoptotic RPEs cells have lost the membrane barrier system. Therefore, AO binds to all acidic portions, independent of the biological proton distribution. The lysosomes of these cells have also lost their acidic fluid through the damaged membrane; therefore, AO leaks from acidic lysosomes and red fluorescent leakage from the lysosomes can be observed [39]. As for the Fig. 5A A2E group, the blue light group and A2E + blue light group showed.

The redistribution of AO in RPEs relied on proton gradients on intact lysosomal membranes [40]. There was a specific functional protein H( +)-ATPase (v-ATPase) on lysosomal membrane, which could keep the inside lysosomal pH value within an acidic region relied on transporting H( +) from cytoplasm to lysosomes utilized ATP produced by hydrolysis [41]. In lysosomes with intact lysosomal membranes, high concentration of AO existed in protonated oligomeric form and appeared red fluorescence, while low concentration of AO existed in deprotonated monomeric form and appeared green fluorescence in nuclei, in damaged lysosomes with decreased or lost proton gradients, or in the cytoplasm [12, 42]. For this reason, AO staining method was used to detect LMP change.

We used LSCM to observe LMP change and analyzed the result quantitatively [43]. The experiment result showed that the mean fluorescence intensity (red) in blue light irradiation group, A2E-loaded group, and blue light irradiation + A2E-loaded group were lower than the value in the control group, which indicated both A2E-loaded and blue light irradiation could induce LMP increasing. Besides, the mean fluorescence intensity (red) in the blue light irradiation + A2E-loaded group was the lowest among the four groups which revealed those two factors had a synergistic effect on LMP increasing than the inference of each factor alone (Fig. 5B).

## Conclusion

In this study, the optimum A2E loading concentration was preliminary determined at 25 μmol/L, and a cell cultured model of A2E-loaded and blue light irradiation inducing RPEs apoptosis in vitro was constructed. The results indicated that both A2E-loaded and blue light irradiation could induce RPEs apoptosis, and the two factors had a synergistic effect. To confirm that LMP increasing could initiate RPEs mitochondrion-dependent apoptotic pathway, we chose the mean red fluorescence intensity as the statistics indicator to monitor LMP change in RPE cells loaded with A2E after blue light irradiation. Through the interference of A2E and blue light, we identified the mean red fluorescence intensity in lysosomes in A2E group and blue light group were decreased compared with control group, which shows both A2E and blue light could increase LMP, resulting AO leaking from lysosomes. In addition, the two factors effect together showed a greater impact on LMP increasing.

These data provided a good experimental foundation of studying the mechanism of RPEs apoptosis loaded with A2E after blue light irradiation in vitro and shed new light on the cause of retinal degenerative diseases in humans.

## Data Availability

The datasets used and analyzed during the current study are available from the corresponding author on reasonable request.
